# Flexible Substrate-Compatible and Efficiency-Improved Quantum-Dot Light-Emitting Diodes with Reduced Annealing Temperature of NiO_x_ Hole-Injecting Layer

**DOI:** 10.3390/molecules29122828

**Published:** 2024-06-13

**Authors:** Shuai-Hao Xu, Jin-Zhe Xu, Ying-Bo Tang, Shu-Guang Meng, Wei-Zhi Liu, Dong-Ying Zhou, Liang-Sheng Liao

**Affiliations:** 1 Institute of Functional Nano & Soft Materials (FUNSOM), Soochow University, Suzhou 215123, China; 2014401012@stu.suda.edu.cn (S.-H.X.); 2014401026@stu.suda.edu.cn (J.-Z.X.); 2014401014@stu.suda.edu.cn (Y.-B.T.); 20214214066@stu.suda.edu.cn (S.-G.M.); 20224014009@stu.suda.edu.cn (W.-Z.L.); lsliao@suda.edu.cn (L.-S.L.); 2 Jiangsu Key Laboratory for Carbon-Based Functional Materials & Devices, Soochow University, Suzhou 215123, China

**Keywords:** flexible displays, quantum dot, nickel oxide, interfacial engineering, hole injection

## Abstract

The growing demand for wearable and attachable displays has sparked significant interest in flexible quantum-dot light-emitting diodes (QLEDs). However, the challenges of fabricating and operating QLEDs on flexible substrates persist due to the lack of stable and low-temperature processable charge-injection/-transporting layers with aligned energy levels. In this study, we utilized NiO_x_ nanoparticles that are compatible with flexible substrates as a hole-injection layer (HIL). To enhance the work function of the NiO_x_ HIL, we introduced a self-assembled dipole modifier called 4-(trifluoromethyl)benzoic acid (4–CF_3_–BA) onto the surface of the NiO_x_ nanoparticles. The incorporation of the dipole molecules through adsorption treatment has significantly changed the wettability and electronic characteristics of NiO_x_ nanoparticles, resulting in the formation of NiO(OH) at the interface and a shift in vacuum level. The alteration of surface electronic states of the NiO_x_ nanoparticles not only improves the carrier balance by reducing the hole injection barrier but also prevents exciton quenching by passivating defects in the film. Consequently, the NiO_x_-based red QLEDs with interfacial modification demonstrate a maximum current efficiency of 16.1 cd/A and a peak external quantum efficiency of 10.3%. This represents a nearly twofold efficiency enhancement compared to control devices. The mild fabrication requirements and low annealing temperatures suggest potential applications of dipole molecule-modified NiO_x_ nanoparticles in flexible optoelectronic devices.

## 1. Introduction

The potential applications of flexible displays in portable and mobile electronics, such as foldable smartphones, healthcare devices, and automotive displays, have attracted significant interest [1]. Quantum-dot light-emitting diodes (QLEDs) have emerged as a promising candidate for advancing flexible display technologies due to their superior luminescence efficiency, narrow spectral linewidth, and tunable emission colors [2,3,4]. Unlike traditional organic light-emitting diodes (OLEDs) that require vacuum deposition techniques [5,6], QLEDs can be efficiently produced using all-solution-based methods, such as inkjet printing [7], spin-coating [8], or blade-coating [9]. More importantly, the solution processability of QLEDs allows for the deposition of quantum-dot (QD) layers onto a wide range of flexible substrates at low temperatures, while simultaneously maintaining high throughput and minimizing material waste. However, flexible QLEDs still encounter several challenges, such as a high processing temperature for charge-injection/-transporting layers, difficulties in achieving uniform quantum-dot layers on flexible substrates, device degradation during bending and stretching, and lack of reliable flexible encapsulation techniques.

In terms of hole-injection/-transporting layers, poly(3,4-ethylenedioxythiophene):polystyrene sulfonate (PEDOT:PSS) is commonly utilized due to its high work function and superior conductivity [10]. However, its susceptibility to moisture absorption and acidic properties can lead to the degradation of the underlying indium tin oxide (ITO) electrode, posing a significant obstacle to the sustained efficient operation of the device.

Recently, non-stoichiometric transition metal oxide, NiO_x_, has attracted extensive attention as a promising hole-injection/-transporting layer in QLEDs [11,12,13,14,15,16,17,18,19,20], OLEDs [21,22], perovskite light-emitting diodes [23,24], and perovskite solar cells [25,26]. The approaches to preparing the NiO_x_ layers include vacuum-based techniques (e.g., sputtering [11,18], thermal evaporation [25], and pulsed-laser deposition [26]) and solution-based methods (e.g., sol-gel [12,13] and solution-combustion [15,21]). However, the vacuum-based processes, which involve costly and complex equipment, as well as the solution-based processes, which require high annealing temperatures (>275 °C) to induce decomposition and crystallization, present challenges in fabrication and hinder the production of flexible QLEDs. As an alternative, the pre-crystallized NiO_x_ nanoparticles (NPs) can be utilized directly to form a uniform thin film at a relatively low annealing temperature (<150 °C) and are compatible with the flexible substrates [27,28,29,30,31,32,33,34,35,36]. However, the electroluminescence performance of QLEDs utilizing NiO_x_ NPs remains unsatisfactory in comparison to those based on PEDOT:PSS due to the inferior hole injection and transportation capability. Extensive research efforts have been dedicated to enhancing the work function and conductivity of NiO_x_ nanoparticles to address these issues. For instance, Lee et al. utilized surface treatment with a dipole molecule 4-(trifluoromethyl)benzoic acid to modify the NiO_x_ NP-based hole-transporting layer (HTL), resulting in a stable high work function of ≈5.5 eV and an external quantum efficiency (EQE) of 18.8% for InP-based QLEDs, comparable to that of the PEDOT:PSS-based device [31]. Zhang et al. demonstrated that the CdS/CdSe/ZnS-based QLED using Fe-doped NiO_x_ NPs as a hole-injection layer (HIL) achieved a maximum current efficiency (CE) of 5.93 cd/A [30]. These positive outcomes confirm the compatibility and the feasibility of NiO_x_ NPs as HTL and/or HIL for efficient and stable QLEDs. However, due to the presence of defective states on the surface of intrinsic NiO_x_ NPs leading to exciton quenching at the NiO_x_ NPs/QDs interface, NiO_x_ NPs are more suitable for functioning as HIL rather than HTL. While the electrical properties of NiO_x_ NPs can be adjusted through ionic doping, precise control of the doping level is crucial as the device performance is highly sensitive to the dopant amount. Therefore, developing efficient un-doped NiO_x_ NP HILs with favorable energy levels using the low-temperature-annealed solution method for QLED applications remains a challenging task.

In this study, we adopt NiO_x_ nanoparticles as the hole-injection layer and utilize a low-temperature annealing method (100 °C) for film preparation. The surface of the NiO_x_ nanoparticles has been modified and the defects have been passivated using the dipole molecule 4-(trifluoromethyl)benzoic acid (4–CF_3_–BA), as confirmed by Fourier-transform infrared (FTIR) and contact angle tests, which validate the successful attachment of dipole molecules. X-ray photoelectron spectroscopy (XPS) characterization of NiO_x_ films before and after modification reveals a significant increase in NiO(OH) and Ni^3+^ components, indicating improved film conductivity. The interaction of dipoles shifts the vacuum level of NiO_x_, reduces hole transport barriers, and promotes efficient light emission in the device. As the 4–CF_3_–BA-modified NiO_x_ is utilized as an HIL in QLEDs, the optimized device demonstrates a significant increase of approximately 200% in maximum current efficiency and peak EQE, achieving values of 16.1 cd/A and 10.3%, respectively. Additionally, the efficiency roll-off with increasing luminance was effectively reduced. Our work advances the understanding of surface/interface interaction mechanisms in optoelectronic devices, laying a foundation for the widespread application of large-area, high-efficiency flexible QLEDs.

## 2. Results

**Figure 1:**
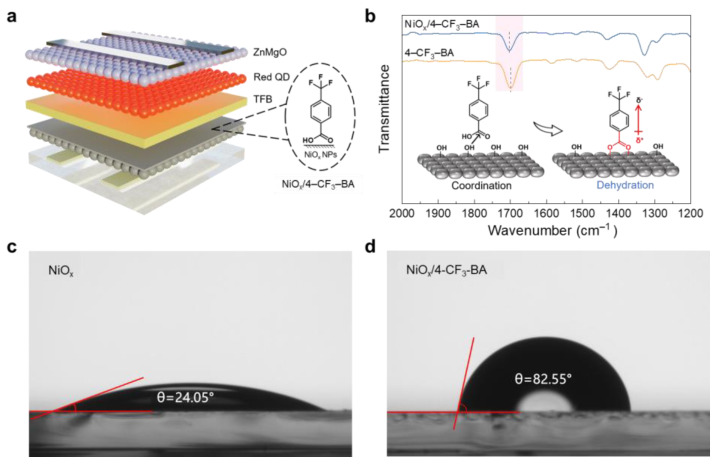
Device structure and dipole molecule orientation: (**a**) Schematic diagram of the QLEDs structure. (**b**) FTIR spectra of the dipole molecule and modified NiO_x_ film (inset: schematic diagram of dipole molecule anchored on the NiO_x_ surface). Contact angles of (**c**) the intrinsic NiO_x_ film and (**d**) the modified NiO_x_ film.

As illustrated in Figure 1a, we prepared the dipole molecules on the NiO_x_ nanoparticle’s surface by the adsorption of 4–CF_3_–BA from ethanol solution. FTIR spectroscopy (Figure S1) was utilized to investigate the potential chemical interactions between the functional groups of the dipole molecule and the NiO_x_ surface. As illustrated in Figure 1b, the NiO_x_/4–CF_3_–BA composite displayed characteristic peaks similar to those of isolated 4–CF_3_–BA molecules, indicating the successful incorporation of 4–CF_3_–BA onto the NiO_x_ surface through spin-coating and subsequent annealing. The vibration mode appearing around 1700 cm^−1^ corresponds to the stretching of the carbonyl group (–C=O) [37]. The coordination of carbonyl oxygen with metal Ni^2+^ (where the lone pair electrons of oxygen occupy the 3D orbital of Ni^2+^) and the subsequent dehydration reaction lead to a higher wavenumber shift in vibrational peak, from 1699.2 cm^−1^ to 1703.1 cm^−1^, enhancing the stability of the functional group. Simultaneously, due to the strong electron-withdrawing capability of the trifluoromethyl group, when connected to a benzene ring with a conjugation effect, the electron cloud distribution tends to be significantly biased toward the trifluoromethyl group [25]. Therefore, as illustrated in the inset of Figure 1b, 4–CF_3_–BA molecules can form a dipole layer on the NiO_x_ surface, with the direction from the carboxyl group pointing toward the trifluoromethyl group, facilitating the conduction of charge carriers.

The integration of dipole molecules was further confirmed by measuring the static contact angle of water on the different NiO_x_ films with or without 4–CF_3_–BA. As shown in Figure 1c,d, after 4–CF_3_–BA modification, the contact angle significantly increases from 20.85° to 82.55°, indicating a reduction in wettability due to the coverage of NiO_x_ with hydrophobic trifluoromethyl-terminated dipoles. The reduced wettability can be attributed to the removal of hydrophilic hydroxyl groups from the NiO_x_ surface due to reactions with the carboxyl group of the 4–CF_3_–BA and subsequent formation of trifluoromethyl-terminated dipoles [12,38], which aligns with the predicted molecular orientation. Given that the subsequently deposited poly(9,9-dioctylfluorene-co-N-(4-(3-methylpropyl)) diphenylamine) (TFB) is dissolved in a non-polar chlorobenzene solvent, increasing the hydrophobicity of the NiO_x_ surface by 4–CF_3_–BA will be beneficial in improving the orientation morphology of the polymer layer, resulting in a more homogeneous HTL.

**Figure 2:**
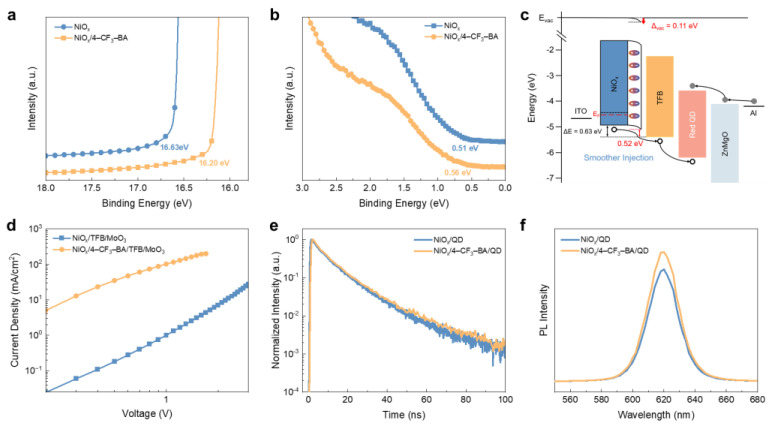
Impact of the dipole layer on band diagram and hole transport performance. UPS spectra depict (**a**) the secondary electron cutoff region and (**b**) the valence band region of intrinsic NiO_x_ and modified NiO_x_ films. (**c**) Schematic diagram of the QLEDs energy levels. (**d**) J–V characteristics double-logarithmic curves of hole-only devices. (**e**) Time-resolved and (**f**) steady-state PL spectra of the QD film with different inserted layers.

To explore the impact of dipole molecule assembly on hole injection, ultraviolet photoelectron spectroscopy (UPS) was conducted to analyze the variations in surface electronic states (Figure S2). The dipole moment of 4–CF_3_–BA, which has separated positive and negative charge centers, is oriented from the carboxyl group toward the para-positioned trifluoromethyl group. When the dipole is directed toward NiO_x_, there is an increase in surface electron density, an elevation in electrostatic potential, and a downward shift in vacuum level. On the other hand, directing the dipole away from NiO_x_ results in an upward shift in vacuum level [38]. Considering the NiO_x_ nanoparticles used in this experiment have a valence band maximum (VBM) above the deep highest occupied molecular orbital (HOMO) of the organic HTL, the dipole directed away from the surface is anticipated to increase the work function of NiO_x_. As shown in the secondary electron cutoff region in Figure 2a, the treatment with 4–CF_3_–BA elevated the work function of NiO_x_ from 4.59 to 5.02 eV. On the other hand, as depicted in the curves in the valence band region (Figure 2b), the energy differences between the valence band maximum (VBM) and the Fermi level are measured at 0.51 eV for the intrinsic case and 0.56 eV for the modified case. Based on the UPS characterization results, the band diagram depicted in Figure 2c illustrates that the insertion of dipole molecules between the NiO_x_ HIL and the TFB HTL causes an upward shift in the vacuum level at the interface, resulting in a smoother alignment of energy levels for hole injection.

The hole injection capability of the NiO_x_ layers was quantitatively evaluated through the fabrication of hole-only devices (HODs) with the structure of “ITO/NiO_x_ (with or without 4–CF_3_–BA)/TFB/MoO_3_/Al” having been fabricated. Figure 2d shows the current density versus voltage characteristics of the HODs in dark conditions. The HOD with the modified NiO_x_ exhibits a significant improvement compared to the control device, with a current density exceeding two orders of magnitude higher at the same bias voltage. Specifically, at 1.0 V, the modified NiO_x_ HOD achieves a current density of 102 mA/cm^2^, while the intrinsic device only reaches 1.0 mA/cm^2^. The increase in hole current density can be attributed to the improved hole injection capability resulting from the reduced energy level offset between the NiO_x_ and TFB layers, as both HODs were prepared using identical structures and methods, except for the presence of the dipole layer.

Time-resolved photoluminescence (TRPL) is a powerful tool to assess radiative and non-radiative recombination processes in bulk or at the interface through analysis of fluorescence lifetimes. In this study, different charge injection layers were inserted at the interface between the quantum dots and the glass to investigate the impact of interface interactions on exciton recombination. As shown in Figure S3, quantum dots coated on the intrinsic NiO_x_ exhibit a faster decay in photoluminescence compared to the QD on glass. This can be attributed to exciton quenching caused by defects on the NiO_x_ surface [31]. Interestingly, the insertion of 4–CF_3_–BA slightly extended the exciton lifetime of the QD film (Figure 2e). The TRPL spectra were analyzed using triexponential models, and the average luminesce lifetimes were calculated using the equation $\tau_{avg}=\left(\sum_{i=1}^{3}B_{i}\tau_{i}^{2}\right)/\left(\sum_{i=1}^{3}B_{i}\tau_{i}\right)$, where *τ_i_* and *B_i_* represent the decay component and their corresponding amplitude coefficients, respectively. The fitting results reveal that, compared to the QDs spin-coated on pristine NiO_x_, the average exciton lifetime of QDs increases from 1.65 to 2.38 ns upon the insertion of 4–CF_3_–BA. The prolonged exciton lifetime, along with the improved steady photoluminescence intensity (Figure 2f), collectively confirm the amelioration of exciton quenching at the NiO_x_ surface due to the passivation of surface defects by the dipole molecules [31].

**Figure 3:**
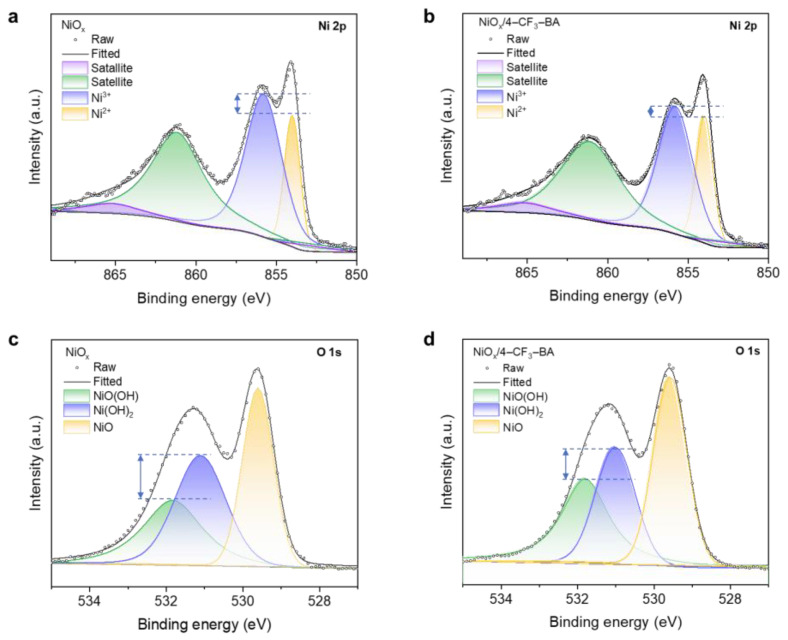
XPS spectra of the NiO_x_ films. Ni 2p_3/2_ spectra of (**a**) the unmodified NiO_x_ film and (**b**) the 4–CF_3_–BA-modified NiO_x_ film. O 1 s spectra of (**c**) the unmodified NiO_x_ film and (**d**) the 4–CF_3_–BA–modified NiO_x_ film.

**Figure 4:**
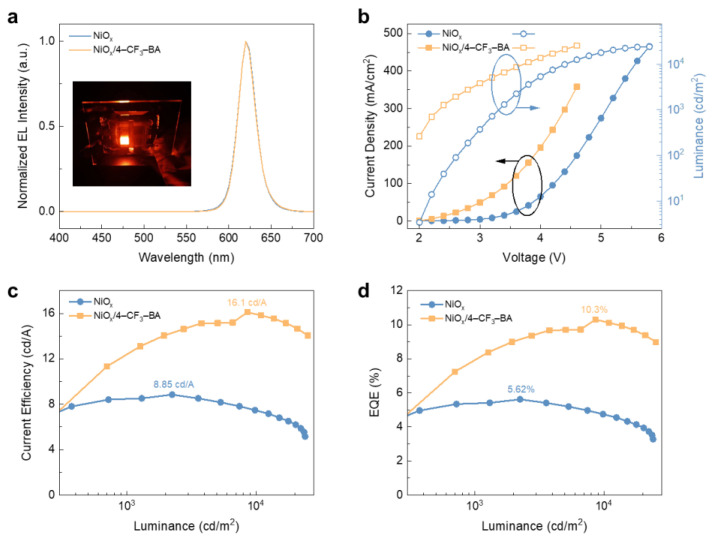
Optoelectronic characteristics of QLEDs: (**a**) Normalized EL spectra (inset: uniformly emitting photo of a flexible QLED with an active area of 100 mm^2^). (**b**) J–V–L, (**c**) current efficiency versus luminance (CE–L), and (**d**) external quantum efficiency versus luminance (EQE–L) characteristics of the NiO_x_-based QLEDs with or without modification.

To understand the defect passivation by the dipole modifier, we explored changes in surface elemental composition and oxidation states of NiO_x_ films before and after dipole molecule modification using X-ray photoelectron spectroscopy (XPS). XPS survey and high-resolution C 1s core level spectra of different NiO_x_ films are shown in Figures S4 and S5, respectively. Figure 3 presents XPS spectra of Ni 2p_3/2_ and O 1s core levels for the bare (Figure 3a,c) and 4–CF_3_–BA-modified (Figure 3b,d) NiO_x_ films [39]. Previous studies have shown that the Ni 2p_3/2_ core level spectra under both conditions exhibit four peaks, corresponding to Ni^2+^ in stoichiometric NiO, Ni^2+^ from Ni(OH)_2_, Ni^3+^ from NiO(OH), and a broad satellite peak [27,39,40]. The O 1s core level spectra also exhibit three peaks, indicating the presence of NiO, Ni(OH)_2_, and NiO(OH). It is worth noting that characteristic peaks corresponding to Ni_2_O_3_ were not observed, as the relatively low annealing temperature (100 °C) during the preparation of the NiO_x_ nanoparticle layers did not provide sufficient energy to facilitate the transformation of the rhombohedral crystal structure of Ni(OH)_2_ into the trigonal hexagonal crystal structure of Ni_2_O_3_. Instead, the formation of NiO(OH) with a rhombohedral structure was observed [39]. This observation is supported by the alignment of the Ni 2p_3/2_ spectra peak position with the standard Ni 2p_3/2_ binding energy reported in the study by Biesinger et al. [35,41]. The relative proportion of the NiO(OH) component, as indicated by the fitted peak area in the Ni 2p_3/2_ spectra, increased from 37.1% to 39.6% following the 4–CF_3_–BA treatment. The increase was also evident in the O 1s spectra, rising from 32.5% for the pristine NiO_x_ to 33.6% for the 4–CF_3_–BA-modified NiO_x_. Previous studies have shown that the presence of NiO(OH) in the film is attributed to the generation of Ni vacancies, with the interchange between Ni^2+^ and Ni^3+^ facilitating hole transport [14]. Consequently, the increased Ni^3+^ component contributes to enhanced conductivity in the NiO_x_ film. Additionally, the production of NiO(OH) alters the vacuum level of the NiO_x_ surface, thereby enhancing hole injection capabilities [22,27,39]. In contrast to commonly reported methods that solely employ UV/ozone or oxygen plasma treatment to increase the NiO(OH) component in NiO_x_ [15,39], our experiment demonstrates that dipole molecules coordinated with the surface can further induce the formation of NiO(OH) on ozone-treated NiO_x_.

Encouraged by the optimized electronic and photophysical properties of the modified NiO_x_, we fabricated all-solution-processed quantum-dot light-emitting diodes (QLEDs) with the structure ITO/NiO_x_/4–CF_3_–BA/TFB/QD/ZnMgO/Al. Simultaneously, a control device (ITO/NiO_x_/TFB/QD/ZnMgO/Al) was prepared under identical conditions for a comprehensive comparison of their electroluminescent (EL) performances. Both devices exhibit pure red emissions with a spectral peak of 620 nm and a narrow full-width half maximum (FWHM) of 23 nm, as illustrated in Figure 4a. The excellent overlap with the photoluminescence (PL) spectral peak of the quantum dots implies that electron-hole pairs mainly recombine within the QD layer (Figure S6). The QLEDs also show stable EL spectra at different driving voltages (Figure S7). The current density-voltage-luminance (J–V–L) characteristics of the devices are illustrated in Figure 4b. The modified NiO_x_-based device exhibits a remarkable increase in current density compared to the intrinsic device. This aligns well with the enhanced hole injection capability observed in hole-only devices, validating the modulation of interface energy levels by dipole molecules. Meanwhile, the modified device requires a lower driving voltage to achieve a certain luminance compared to the control device. Specifically, in the NiO_x_ HIL device, the driving voltages required to attain the luminance of 1000 cd/m^2^ and 10,000 cd/m^2^ are 3.3 V and 4.4 V, respectively, whereas, for the optimized NiO_x_/4–CF_3_–BA device, these voltages drop to below 2.3 V and below 3.7 V. Moreover, the modified device consistently outperforms the control device in luminance throughout the entire range of tested voltages. The increased hole injection efficiency has a significant enhancement effect on such hole mobility-limited light-emitting devices. The optimization of the balance between electron and hole injection is ultimately reflected in the enhancement of device efficiency. As shown in Figure 4c,d, the maximum current efficiency (CE) and peak external quantum efficiency (EQE) of the optimized device reach 16.1 cd/A and 10.3%, respectively, compared to 8.85 cd/A and 5.62% for the intrinsic device, representing a nearly 200% improvement. This advancement is attributed to enhanced hole injection and suppression of exciton quenching at the metal oxide/TFB HTL interface. Additionally, with the increasing brightness, the efficiency roll-off in the 4–CF_3_–BA-modified device is also mitigated. The low annealing temperature (100 °C) of NiO_x_ nanoparticles makes the preparation of flexible devices possible. Using poly(ethylene terephthalate) (PET)/ITO as a substrate, we prepared a 1 cm × 1 cm flexible red QLED with 4–CF_3_–BA-modified NiO_x_ NPs as HIL. The device exhibited bright and uniform electroluminescence (the inset of Figure 4a), confirming the compatibility and feasibility of NiO_x_ NPs as HIL for efficient and flexible QLEDs.

## 3. Materials and Methods

### 3.1. Preparation of NiO_x_ Films

The ethanol solution of NiO_x_ nanoparticles (Avantama, P-21, 2.5 wt%, Stäfa, Switzerland) was diluted to 0.15 wt%. Following filtration through a 0.45 μm polyvinylidene fluoride membrane, the solution was spin-coated onto the patterned ITO glass substrates at 3000 rpm for 30 s, followed by annealing at 100 °C for 10 min. The NiO_x_ film was treated by UV/ozone for 30 min. Then, 5 mg of 4–CF_3_–BA (Aladdin Chemical Ltd., Shanghai, China) was dissolved in 2 mL of anhydrous ethanol (2.5 mg/mL) to prepare the precursor solution. After filtration of the precursor through a 0.45 μm polyvinylidene fluoride membrane, 300 μL of the solution was dropped onto the NiO_x_-coated substrate and immersed for 2 min, followed by spin-coating at 5000 rpm for 20 s and annealing at 100 °C for 5 min. After cooling, the film was rinsed with anhydrous ethanol during spin-coating at 5000 rpm for 20 s to remove molecules not bonded to NiO_x_, followed by annealing at 100 °C for 5 min.

### 3.2. QLEDs Fabrication

The substrate coated with NiO_x_/4–CF_3_–BA was transferred to a glovebox filled with N_2_ for subsequent film preparation. A solution of TFB in chlorobenzene (8 mg/mL) was filtered through a 0.45 μm polyvinylidene fluoride membrane and spin-coated at 3000 rpm for 40 s. The film was annealed at 120 °C for 15 min. CdSe quantum dots (20 mg/mL, Poly OptoElectronics Co. Ltd., Xi’an, China) were diluted to 15 mg/mL in octane solvent, spin-coated onto the TFB layer (2000 rpm, 40 s) followed by annealing at 100 °C for 5 min. A dispersion of ZnMgO nanoparticles (30 mg/mL) was spin-coated at 3000 rpm for 40 s, followed by annealing at 100 °C for 15 min. Finally, the substrate coated with various functional layers was transferred to a thermal evaporator and deposited with a patterned 80 nm Al cathode on top of ZnMgO NPs at a rate of 3 Å/s under vacuum pressure less than 4 × 10^−^^6^ Torr. The QLEDs fabricated on the ITO-coated PET plastic substrates had the same procedure as the ones on glass substrates.

### 3.3. Fabrication of Hole-Only Device

The device structure consists of ITO/NiO_x_ (with or without 4–CF_3_–BA)/TFB/MoO_3_/Al, where the preparation of modified NiO_x_ and TFB layer mirrors the methods mentioned in the fabrication steps of QLEDs. Then, the films are transferred to a thermal evaporator where 7 nm of MoO_3_ is deposited at a rate of 0.3 Å/s under a vacuum pressure less than 4 × 10^−6^ Torr, followed by the deposition of 80 nm Al cathode.

### 3.4. Thin Film and Device Characterization

XPS and UPS measurements were conducted using KRATOS AXIS Ultra DLD from KRATOS Analytical (Manchester, UK). UPS was performed with a He I source (*hν* = 21.22 eV). The samples were prepared on silicon and measured under −5 V bias. Contact angles were measured using an OCA15 from Dataphysics (Filderstadt, Germany). The steady-state and time-resolved photoluminescence (TRPL) were measured with a fluorescence lifetime spectrometer (Quantaurus-τ from Hamamatsu-Photonics K.K., Shimokanzo, Iwata City, Shizuoka Pref., Japan). The steady-state and time-resolved PL measurements were conducted under the excitation of a pulse laser, with an excitation wavelength of 375 nm and a pulse period of 50 ps. FTIR spectra using attenuated total reflectance mode were obtained in the range of wavenumber from 4000 to 400 cm^−1^ during 32 scans, with 2 cm^−1^ resolution (VERTEX 70v Fourier Transform Infrared Spectrometer from Bruker, Billerica, MA, USA). The electroluminescence spectra and luminance were collected by PR-665 (Photo Research Inc., Chatsworth, CA, USA), while the current density and voltage data for both QLEDs and HODs were recorded using a Keithley 2400 (Beaverton, OR, USA) source measuring unit.

## 4. Conclusions

We present a strategy to enhance the work function of NiO_x_ nanoparticles while diminishing the hole transport barrier. Simultaneously, the modification achieves the passivation of interface defects, suppressing the occurrence of exciton quenching. Surface modification with 4–CF_3_–BA can also change the wettability of the substrate surface by replacing –OH terminal groups with aromatic molecular units. Anchoring points are provided by hydroxyl dangling bonds on the metal oxide surface, while the coordination-dehydration reaction between carbonyl oxygen and Ni^2+^ ions promotes the robust assembly of dipole molecules with ideal orientation. This phenomenon is corroborated by the shift in characteristic vibrational peaks in FTIR and the increase in the contact angle. The reduction in the hole injection barrier allows the modified devices to achieve the same luminance as the intrinsic devices at lower driving voltages. Additionally, balanced electron and hole injection enables devices based on modified NiO_x_ HIL to exhibit a maximum CE of 16.1 cd/A and a peak EQE of 10.3%, over three times higher than intrinsic devices, confirming the pivotal role of interface dipoles. The adoption of mild and cost-effective fabrication conditions, coupled with the relatively high efficiency after optimization, offers promising prospects for the widespread application of red QLEDs in large-area flexible optoelectronic devices and emerging fields like virtual reality (VR) and augmented reality (AR).

## Data Availability

The data presented in this study are available on request from the corresponding author. The data are not publicly available due to [insert reason here their containing information that could compromise the privacy of research participants].

## Supplementary Materials

The following supporting information can be downloaded at: https://www.mdpi.com/article/10.3390/molecules29122828/s1, Figure S1: FTIR spectra of NiO_x_ with organic dipole molecule; Figure S2: Full-spectrum diagram of UPS; Figure S3: Time-resolved PL spectra for NiO_x_/QD with and without dipole molecule; Figure S4: Full-spectrum diagram of XPS; Figure S5: C 1 s core levels of NiO_x_ films before and after modifications; Figure S6: Normalized EL and PL spectra of modified devices; Figure S7: EL spectra at different voltages of Control and target samples; Table S1: Fractional peak results of XPS.
