# Supplementary material for: Flexible Substrate-Compatible and Efficiency-Improved Quantum-Dot Light-Emitting Diodes with Reduced Annealing Temperature of NiOx Hole-Injecting Layer
Source: Molecules. 2024 Jun 13;29(12):2828. doi: 10.3390/molecules29122828 (PMC11206919; doi:10.3390/molecules29122828)
Supplement: Supplementary file 1 [file molecules-29-02828-s001.zip › molecules-3016403-supplementary.pdf]

Supporting Information for

Flexible Substrate–Compatible and Efficiency–Improved Quantum-Dot Light-Emitting Diodes with Reduced Annealing Temperature of NiO_x Hole-Injecting Layer

Shuai-Hao Xu [+], Jin-Zhe Xu [+], Ying-Bo Tang, Shu-Guang Meng, Wei-Zhi Liu, Dong-Ying Zhou,[*] Liang-Sheng Liao

Institute of Functional Nano & Soft Materials (FUNSOM), Jiangsu Key Laboratory for Carbon-Based Functional Materials & Devices, Soochow University, Suzhou, 215123 Jiangsu, China

[+] These authors contributed equally to this work.

[*] E-mail: dyzhou@suda.edu.cn

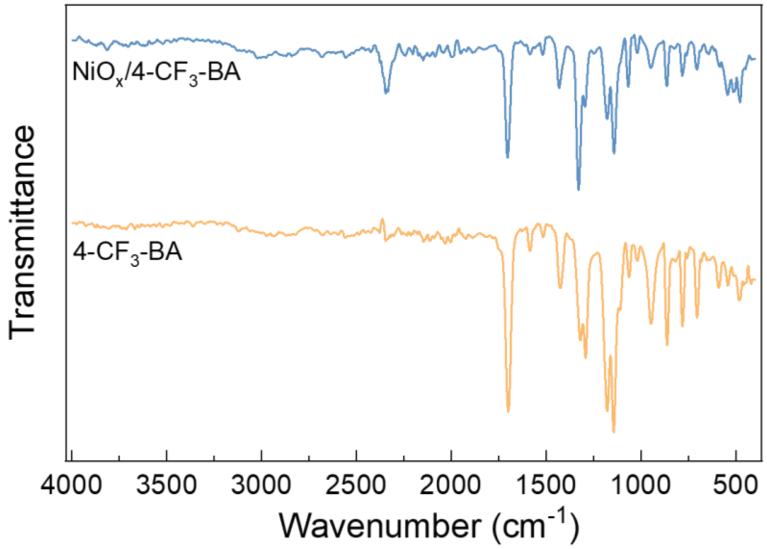

Figure S1. FTIR spectra of NiO_x with organic dipole molecule.

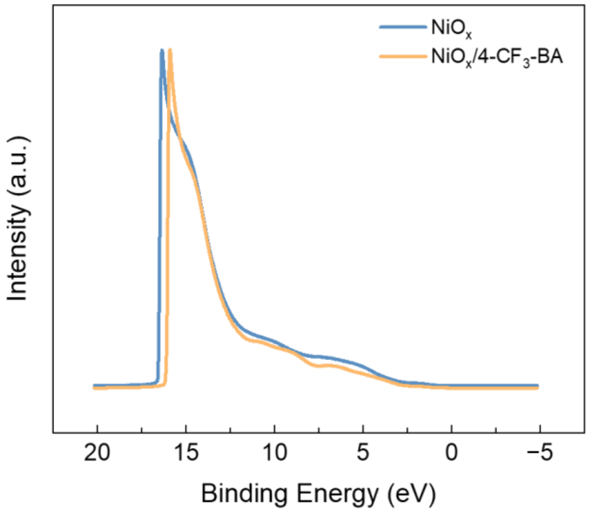

Figure S2. Full spectrum diagram of UPS.

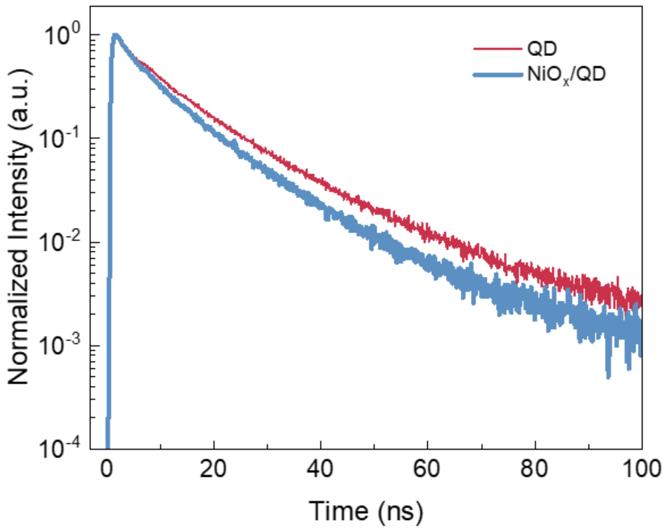

Figure S3. Time-resolved PL spectra of QDs coated on glass and NiO_x.

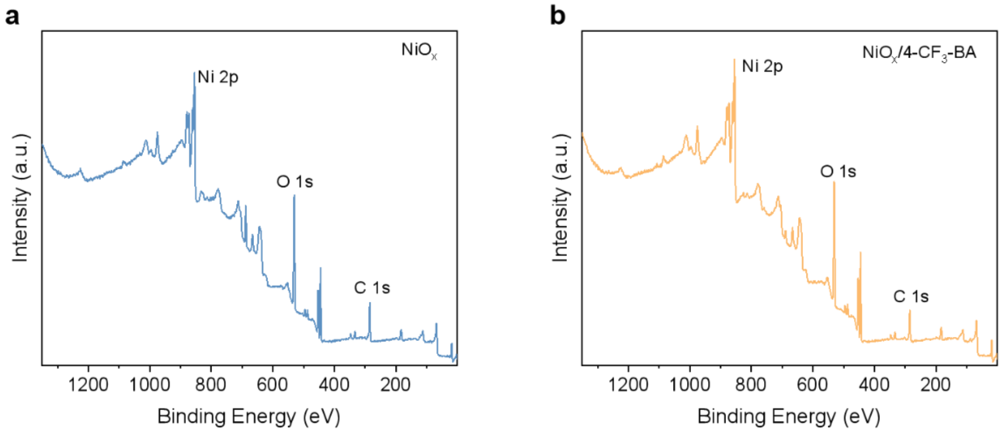

Figure S4. Full spectrum diagram of XPS.

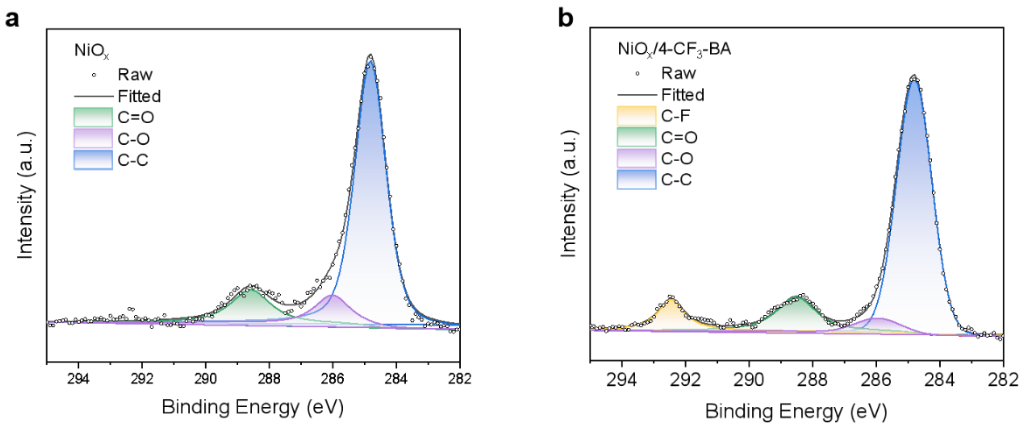

Figure S5. C 1s core levels of NiO_x films before and after modifications.

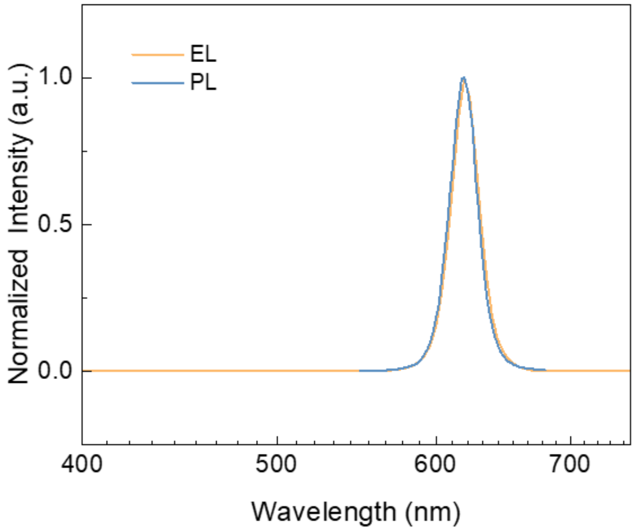

Figure S6. Normalized EL and PL spectra of QDs.

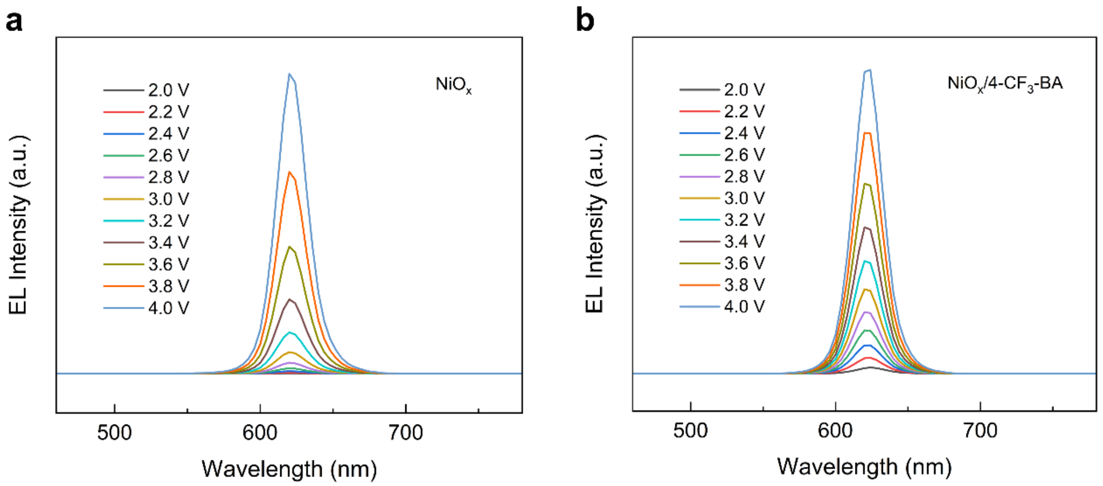

Figure S7. (a) EL spectra of the QLED with NiO_x as HIL under different driving voltages. (b) EL spectra of the QLED with NiO_x/4-CF_3-BA as HIL under different driving voltages.

Table S1. Fractional peak results of XPS.

Peak		Binding Energy (eV)	Peak Area Proportion (%)
Ni 2p (NiO_x)	Ni Satellite1	865.19	5.49
	Ni Satellite2	861.09	41.25
	Ni^{3+}	855.79	37.12
	Ni^{2+}	853.97	16.14
Ni 2p (NiO_x/4-CF_3-BA)	Ni Satellite1	865.19	4.88
	Ni Satellite2	861.32	42.10
	Ni^{3+}	855.89	39.60
	Ni^{2+}	854.08	13.42
O 1s (NiO_x)	NiO(OH)	531.83	32.53
	$Ni(OH)_2$	531.1	35.03
	NiO	529.59	32.44
O 1s (NiO_x/4-CF_3-BA)	NiO(OH)	531.8	33.64
	$Ni(OH)_2$	531.01	29.03
	NiO	529.57	37.34
C 1s (NiO_x)	C=O	288.58	13.79
	C-O	286.02	10.58
	C-C	284.82	75.63
C 1s (NiO_x/4-CF_3-BA)	C-F	292.46	9.8
	C=O	288.52	14.74
	C-O	285.96	4.63
	C-C	284.81	70.82
